# An *in vitro* model using spheroids-laden nanofibrous structures for attaining high degree of myoblast alignment and differentiation

**DOI:** 10.7150/thno.53928

**Published:** 2021-01-16

**Authors:** Miji Yeo, SooJung Chae, GeunHyung Kim

**Affiliations:** 1 Department of Biomechatronic Engineering, College of Biotechnology and Bioengineering, Sungkyunkwan University (SKKU), Suwon 16419, South Korea.; 2 Biomedical Institute for Convergence at SKKU (BICS), Sungkyunkwan University, Suwon 16419, South Korea.

**Keywords:** spheroid, electrospinning, myogenesis, *in vitro* model.

## Abstract

A spheroid is an aggregation of single cells with structural and functional characteristics similar to those of 3D native tissues, and it has been utilized as one of the typical *in vitro* three-dimensional (3D) cell models. Scaffold-free spheroids provide outstanding reflection of tissue complexity in a 3D *in vivo*-like environment, but they can neither fabricate realistic macroscale 3D complex structures without avoiding necrosis nor receive direct external stimuli (i.e., stimuli from mechanical or topographical cues). Here, we propose a spheroid-laden electrospinning process to obtain *in vitro* model achieved using the synergistic effect of the unique bioactive components provided by the spheroids and stimulating effects provided by the aligned nanofibers.

**Methods:** To show the functional activity of the spheroid-laden structures, we used myoblast-spheroids to obtain skeletal muscle, comprising highly aligned myotubes, utilizing an uniaxially arranged topographical cue. The spheroid-electrospinning was used to align spheroids directly by embedding them in aligned alginate nanofibers, which were controlled with various materials and processing parameters.

**Results:** The spheroids laden in the alginate nanofibers showed high cell viability (>90%) and was compared with that of a cell-laden alginate nanofiber that was electrospun with single cells. Consequently, the spheroids laden in the aligned nanofibers showed a significantly higher degree of myotube formation and maturation.

**Conclusion:** Results suggested that the *in vitro* model using electrospun spheroids could potentially be employed to understand myogenic responses for various *in vitro* drug tests.

## Introduction

A spheroid is a three-dimensional (3D) globular cell aggregate, which has emerged as a cell-block unit in tissue engineering. The spheroids, compared to single cells cultured in 2D conditions, are able to express enhanced cellular activities that are conjugated with the release of various signaling factors 1, 2. Specifically, those comprising mesenchymal or adipose stem cells show enhanced neurodifferentiation and osteodifferentiation and up-regulated expression of biochemical components such as enzymes 3-5. In the perspective of engineering a 3D niche in macroscale or a heterogeneous cell complex (vascularized tissue), the use of spheroids has shown benefits of reproducing the structural and functional characteristics of native tissues 6, 7.

Controlling the morphology of spheroids into a native tissue-like structure or an aligned elongated structure is another important factor that promotes cell growth and differentiation. For instance, human iPS-derived muscle cells were developed in an aligned muscle bundle using pillar structures to interact with a neural stem spheroid 8, 9. In another study, an endothelial spheroid was guided to sprout along the fibrin supporting structure 10. Meanwhile, transformation of a spheroid into a completely elongated aligned structure has not been thoroughly investigated. In this regard, the assessment of cell morphology under tailored conditions (i.e. spheroid-to-spheroid distance, aligned pattern, etc.) would provide a cue to control spheroid alignment.

The fabrication of aligned micro/nanopatterns has been one of the major objectives of tissue regeneration, and numerous methods have been employed 11-13. For instance, it was suggested that wet electrospinning, with a rotating drum, could be used to obtain aligned nanofiber yarns (NFYs) 14, 15, The C2C12 murine myoblast, PC12, and dorsal root ganglia cells were cultured on NFYs, and over 90% cells aligned at 10^o^ within 7 days. Conversely, NFYs were used as sacrificial templates for development of an extracellular matrix (ECM) scaffold with aligned microchannels 16. This enabled not only reconstruction of a tailored 3D macro/micro-structure, but also *in vivo* regeneration. In addition, C2C12 cells were completely aligned within a tailored 3D structure by cell-printing, assisted with aligned gold (Au) nanowires (GNWs) 17. These highly aligned GNWs facilitated development of myotubes into multi-nucleated elongated structures and significantly reduced fibrosis *in vivo* (<10%), as compared with fibrosis reduced by the control without GNWs (40%). In short, the aligned micro/nanopattern is beneficial for regenerating tissues, and aligning spheroids would be the next step in improving cell-to-cell/matrix interactions.

Cell electrospinning (CE) enables direct encapsulation of cells into micro/nanofibers. This typical structure shows advantages of enlarged 3D nutrient accessibility, presence of a fabricated nanofibrous structure similar to ECM, and enhanced cell-to-cell/matrix interactions 18. The first concept of CE that was proposed using a core-shell nozzle supplemented with biosuspension in the inner needle and polydimethylsiloxane in the outer needle 19, 20. The genuine viability and proliferation of astrocytoma and smooth muscle cells showed the possibilities of utilizing CE. For another application of CE, we previously developed the process of CE combined with topographical cues that can induce alignment of electrospun (e-spun) cells by controlling the direction of nanofibers 21. Here, we demonstrated the anisotropically stretched morphology of C2C12 cells and their differentiation into myotubes, revealing the potential in guiding cell morphology and growth. However, the dispersed single cells required a long culturing period to be able to participate in cell-to-cell interactions, and hence, we needed to resolve the low efficiency in cell-to-cell interactions.

To overcome the limitation of the e-spun cell structure, spheroid-electrospinning was utilized to fabricate a spheroid-laden micro/nanofibrous bundle with a topographical cue. The synergistic effects obtained using an aligned cue in a precise fibrous structure and cell-aggregation could promote proficient elongation and differentiation of the e-spun myoblast-spheroids into a 3D structure. To assess spheroid morphogenesis on an aligned cue, the spheroids were placed at different distances (100~600 μm) on e-spun fibers and evaluated the distance most favorable for spheroid alignment and maturation. After optimizing processing conditions, such as an electric field and the nozzle-to-electrode distance, the C2C12-spheroids were e-spun on a microscale polycaprolactone (PCL) strut to mechanically support the soft e-spun fibers to develop into an aligned spheroid fibrous bundle. The spheroids could appropriately align myoblasts along the nanosized fibrous structures on the PCL strut, resulting in the stable construction of myofibers. Based on the various cellular activities, the e-spun spheroids could clearly give not only skeletal muscle-specific biochemical cues that induce the maturation of skeletal cells, but also fibrous topographical cues that induced well-organized myotube formation.

## Materials and Methods

### Cell culture

C2C12 myoblasts (ATCC, Manassas, VA, USA) were cultured in Dulbecco's Modified Eagle's Medium high glucose (DMEM high glucose; Sigma-Aldrich, St. Louis, USA), containing 10% fetal bovine serum (FBS; BIOWEST, MO, USA) and 1% penicillin/streptomycin (PS; Hyclone, Logan, UT, USA). Human umbilical vein endothelial cells (HUVECs) were cultured in Endothelial Cell Basal Medium-2 (EBM-2; CC-3156; Lonza, Basel, Switzerland) supplemented with Endothelial Cell Growth Medium-2 SingleQuots (EGM-2; CC-4176; Lonza, Basel, Switzerland) and 1% antibiotic. The myoblasts and HUVECs were incubated at 37 ^o^C in 5% CO_2_, and the medium was changed every 2 d.

Following the reported protocol 22, human muscle progenitor cells (hMPCs) were isolated from human muscle musculus gracilis muscles (from 51-64 year-old women, de-identified). The biopsied muscles were digested in DMEM containing 0.4 wt% dispase (Sigma-Aldrich, St. Louis, USA) and 0.2 wt% type I collagenase (Worthington Biochemical, USA) at 37 ^o^C for 2 h. After filtering using a strainer (100-mm pore), the specimens were centrifuged for 5 min at 1500 rpm. The pellet was suspended in DMEM/F12 containing FBS (18%), dexamethasone (0.4 mg/mL), human insulin (10 mg/mL), human basic fibroblast growth factor (hbFGF; 1 ng/mL), and human epidermal growth factor (hEGF; 10 ng/mL), and incubated on a plate coated with collagen type I (1 mg/mL; MSBio, South Korea) in the humidified atmosphere (5% CO_2_) at 37 ^o^C overnight. After 8-10 d, the cells were sub-cultured using DMEM containing 2% chicken embryo extract (Gemini Bio-Products, USA), 20% FBS, and 1% PS. The medium was changed every 2 d, and the cells were used up to passage 4-5 for the experiments.

### Fabrication of spheroids

The spheroids were fabricated using micro-mold spheroid kits (3D Petri Dish; MicroTissues, Sharon, MA, USA), following the manufacturer's protocol. Briefly, agarose micro molds, constructed in a 3D Petri Dish, were equilibrated with cell culture medium, and the medium was then removed. Thereafter, C2C12 or HUVEC cells (3.2 × 10^4^) were casted onto each agarose mold and cultured in growth medium (2.5 mL) to fabricate C2C12 or HUVEC spheroids (100 µm). After 3 d of aggregation, the fabricated spheroids were harvested. The formation of spheroids was captured with a digital camera connected to an optical microscope (BX FM-32, Olympus, Japan). To measure the diameter of the spheroid, the captured images were analyzed using the Image J software.

### Fabrication of a grooved structure on a fibrous mat by alginate printing

The aligned fibrous mat was fabricated using a mixture of 2-wt% alginate (LF10/50; FMC BioPolymer, Drammen, Norway) and 3-wt% polyethylene oxide (PEO; Sigma-Aldrich, St. Louis, USA) in triple-distilled water. The electrospinning conditions [0.075 kV/mm (10.5-kV high voltage direct current and 140-mm electrode-to-collector distance); 0.25 mL/h; 3 min] were selected from a previous study 21. The flow rate of the solution was controlled using a syringe pump (KDS 230; Samwon, Busan, South korea) and the electric field was produced using a power supply (SHV300RD-50K, Concertech, Seoul, South Korea). The alginate fibers were deposited between parallel electrodes for alignment, and 4.5-wt% alginate in triple-distilled water was extruded onto the aligned alginate fibrous mat in the low-temperature stage applied using a 3D printing system. The fibrous alginate mat was placed in the temperature controllable stage (-18 ^o^C), the alginate solution was extruded using a 3D robot (DTR3-2210-T-SG, DASA Robot, Bucheon, South Korea) through a nozzle (100 µm) along the direction of alignment. The printed structure was then crosslinked with 2-wt% CaCl_2_. To adjust the distance between spheroids, different density spheroids were seeded (100 μm: 1.9 × 10^5^/mL; 200 μm: 1 × 10^5^/mL; 300 μm: 6 × 10^4^/mL; 450 μm: 4 × 10^4^/mL).

### Preparation of bioink for cell/spheroid electrospinning

The alginate-based solution was prepared by dissolving 2-wt% alginate and 3-wt% PEO in triple-distilled water. The components were stirred and homogeneously mixed using a magnetic bar for 48 h at room temperature. Then, to prepare a cell-laden bioink, C2C12 or HUVEC cells (5 × 10^6^) were isolated and added to the alginate solution (1 mL). Meanwhile, to prepare spheroid-laden bioink, 40,000 C2C12 or HUVEC spheroids, equivalent to 5 × 10^6^ cells, were harvested and mixed with the solution (1 mL).

The storage modulus (*G'*) and complex viscosity (η^*^) of alginate (2 wt%)/PEO (2, 3, 4 wt%) solutions were analyzed using a rotational rheometer (Bohlin Gemini HR Nano; Malvern Instruments, Surrey, UK). Using a cone-and-plate geometry (4° cone angle, 40mm diameter, and 150 μm gap), a stress sweep (from 0.1 to 100 Pa) was recorded in constant frequency of 1 Hz at 23 °C. Hereafter, the mixture of 2-wt% alginate and 3-wt% PEO was selected to proceed the study.

The surface tension was measured by capillary-rise method in the liquid-air surface. After a glass capillary was dipped in the solution of alginate/PEO for 1 h, the capillary was captured using a digital camera. Then, the contact angle and rises of solution in the capillary were measured. Also, the electric conductivity was evaluated by a pH/conductivity meter (Consort C861; Consort bvba, Turnhout, Belgium) (n = 3).

### Sedimentation of spheroids in bioink

The spheroids in different diameters (100, 200, and 300 mm) were laden in the 1 mL bioink and put into a 1.5 mL microtube. The images were captured depending on the time (*in situ*, 2 h, and 6 h) using a digital camera, and analyzed using the Image J software. After converting the images into gray scale, the sedimented spheroids in white color were quantitatively represented.

### Scaffold fabrication

PCL (M_w_ = 45,000; Sigma-Aldrich, St. Louis, USA) was used to fabricate a PCL strut (diameter = 300 μm) with a melt-plotting system. Melted PCL was printed through a metal nozzle (diameter = 300 μm) using pneumatic pressure (340 kPa).

For sample fabrication, electrospinning was performed using bioinks laden with cells/spheroids. To optimize the electrospinning conditions, various electric field (0.05, 0.075, 0.1, and 0.125 kV/mm) and various nozzle-to-electrode distances (80, 100, 120, and 140 mm) were used. After investigation of electrospinning parameters, the conditions (nozzle-to-electrode distance = 140 mm; electrode-to-electrode distance = 30 mm; electric field = 0.075 kV/mm; electrospinning time = 3 min) were selected. The experiments were performed at 23 °C in approximately 40% humidity. After 2 min of crosslinking with 2 wt% CaCl_2_ (Sigma-Aldrich, St. Louis, USA), the samples were immersed in DMEM to obtain cultures.

### Scaffold characterization

To obtain scanning electron microscopy (SEM; SNE-3000M; SEC Inc., Suwon, South Korea) images, the samples laden with cells/spheroids were fixed in 2.5% glutaraldehyde for 4 h. After rinsing the samples in 50%, 70%, 80%, 95%, and 100% alcohol for 10 min each, they were treated with hexamethyldisilazane for 1 h. The samples were air dried and coated with Au. The images were captured using SEM and the orientation of the fibers were analyzed using the Image J software.

The tensile modulus was measured using a microtensile tester (Toptech 2000; Chemilab, Suwon, South Korea). The samples were freeze dried for the test, and uniaxial stretching (0.05 mm/s) was applied to obtain a stress-strain curve (n = 5).

### Assessment of *in vitro* cellular activities

After 1 and 7 d of culture, the e-spun cells/spheroids were treated with 0.15 mM calcein AM and 2 mM ethidium homodimer-1 for 30 min in an incubator. To measure cell viability, the stained scaffolds were observed with a confocal laser scanning microscope (LSM 700; Zeiss, Oberkochen, Germany) (n = 5). Image J software was used for analysis of cell viability, which was calculated by (the number of live cells/the total number of cells×100%). Here, a spheroid was assumed as 125 cells.

To count electrospun cell/spheroid numbers, the samples were treated with trypan blue solution (1:100 in TBS; Sigma-Aldrich, St. Louis, USA). The fibers were captured using an optical microscope and analyzed using the Image J software. A spheroid was counted as 125 cells.

The cell proliferation rate was measured using a CCK8 assay (Cell Counting Kit-8, Dojindo Molecular Technology, Japan) (n = 6). The cell/spheroid-laden samples with 300 μm diameter and 10 mm length were prepared. After 1, 7, and 14 d of culture, the samples were placed in a new plate and washed with tris-buffered saline (TBS) twice. DMEM (200 μL), added to CCK8 solution (20 µL), was applied to the samples at 37 °C in 5% CO_2_ for 4 h. The values of optical density (λ_450nm_) were normalized with respect to the values at 1 d.

To evaluate cell morphology and myogenic and angiogenic differentiation, diamidino-2-phenylindole (DAPI)/phalloidin, myosin heavy chain (MHC), sarcomeric α-actin, cluster of differentiation 31 (CD31/platelet-endothelial cell adhesion molecule (PECAM-1)), and vascular endothelial (VE)-cadherin staining were performed. The samples were rinsed with TBS twice and fixed with 3.7% formaldehyde solution at 4 °C for 12 h. For DAPI/phalloidin staining, the samples were stained with DAPI (Invitrogen, Carlsbad, CA, USA) and phalloidin (Invitrogen, Carlsbad, CA, USA) conjugated with Alexa Fluro 568. For DAPI/MHC and sarcomeric α-actin staining, the fixed samples were permeabilized with 0.3% Triton X-100 for 10 min and immersed in 1% bovine serum albumin (BSA; Sigma-Aldrich, St. Louis, USA) for 1 h. Then, the samples were treated with rabbit anti-MHC antibody (1:200 in TBS), anti-sarcomeric α-actin antibody (1:200 in TBS), anti-CD31 antibody (1:200 in TBS) or anti-VE-cadherin antibody (1:200 in TBS) at 4 °C overnight. Secondary antibody conjugated with Alexa Fluor 488 or 594 and DAPI were added to the samples, which were then placed in an incubator for 1 h. The fluorescent images were captured using a confocal laser scanning microscope.

### Real-time reverse transcription polymerase chain reaction (RT-PCR)

The expression levels of myokines [interleukin (IL)-6, tumor necrosis factor-α (TNF-α), monocyte chemoattractant protein 1 (MCP-1), chemokine (C-X-C motif) ligand 1 (CXCL1), and chemokine (C-C motif) ligand 5 (CCL5)] and myogenic specific genes [MyoD, myogenin (MyoG), and MHC 2 (Myh2)] were quantitatively measured by RT-PCR. The values of myokines were normalized by s29, and the values of myogenic genes were normalized by beta actin. In brief, the TRI agent (Sigma-Aldrich) was used to isolate RNA from the cultured sample. The purity and concentration were evaluated using a spectrophotometer (FLX800T; Biotek, Winooski, VT, USA). The cDNA was synthesized from RNase-free DNase-treated total RNA using a reverse transcription system. Thunderbird SYBR qPCR mix (Toyobo, Osaka, Japan) was applied to the cDNA, and Taqman assays (Thermo Fisher Science, MA, USA) were performed. The samples were then analyzed using the StepOnePlus Real-Time PCR System (Applied Biosystems, Forster City, CA, USA), according to the manufacturer's protocol.

### Statistical analysis

The data are presented as mean ± standard deviation. The analysis was performed using SPSS 18 software (SPSS, Inc., IL, USA). The independent-sample t-test was used for comparison between two groups. The analysis of variance (ANOVA) was used for comparison among multiple groups. The statistical significance is indicated as p* <0.05, p** <0.01, and p*** <0.001.

## Results & Discussion

### Preparation of spheroids using an agarose mold

**Figure 1:**
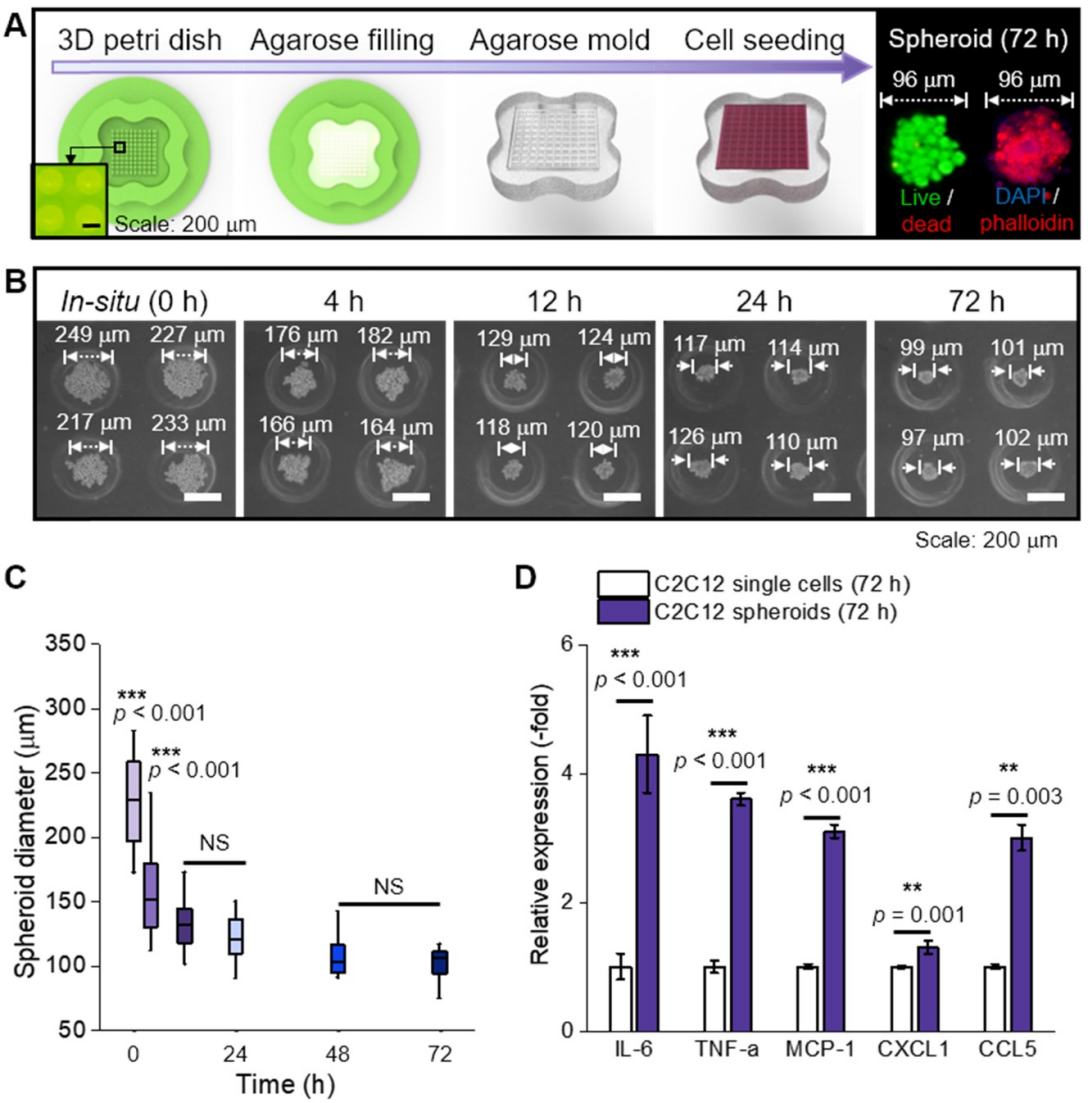
(A) Schematic representation of spheroid fabrication; (B) optical images of spheroid formation for 72 h; (C) The change in spheroid diameter (n = 50); (D) Relative gene expression of C2C12 single cells and spheroids (n = 5).

In this study, C2C12 cells (125 cells/spheroid) were aggregated into a spheroid by seeding them onto an agarose mold (Figure 1A). The progress of cell aggregation is presented by optical images (Figure 1B); the diameter of the spheroids continuously decreased from 249 μm to 99 μm with increasing the culture period (Figure 1C). Since the spheroid diameter remained constant at 99 ± 2 μm past the 72 h timepoint, 72 h was selected as the culturing period of the spheroid in this study.

In general, cytokines (myokines) are a large family of proteins or polypeptides, which mediate intercellular communication and various cellular activities including cell proliferation and differentiation 23, 24. In respect to a cellular activity regulator, the cytokines of the spheroids were compared to those of the single cells cultured in a dish for 72 h (Figure 1D) 25, 26. RT-PCR was performed to measure several myokines 27-31, such as interleukin (IL)-6 (muscle contraction regulator), tumor necrosis factor-α (TNF-α; pro-inflammatory cytokine), monocyte chemoattractant protein 1 (MCP-1; chemokine that recruits monocytes to the site of vessel injury), chemokine (C-X-C motif) ligand 1 (CXCL1; myogenesis promoting chemokine), and chemokine (C-C motif) ligand 5 (CCL5; chemokine that recruits macrophages that promote myoblast proliferation). As shown, all myokines of C2C12 spheroids were upregulated compared to those of the single cells. We believe that these biochemical components derived from the spheroids could meaningfully affect various cellular activities, such as myoblast growth and formation of myotubes 32. In addition, after the single cells and spheroids were electrospun and cultured for 28 d, all myokines were increased on spheroids (Figure S1A).

### Selection of spheroid size and density for electrospinning

In general, the fabrication of micro/nanofibers homogeneously distributed with microscale particles has been difficult. The reason is that the sedimentation of the particles in the electrospinning solution can be occurred by a low viscose solution and gravitational force of microscale particles.

**Figure 2:**
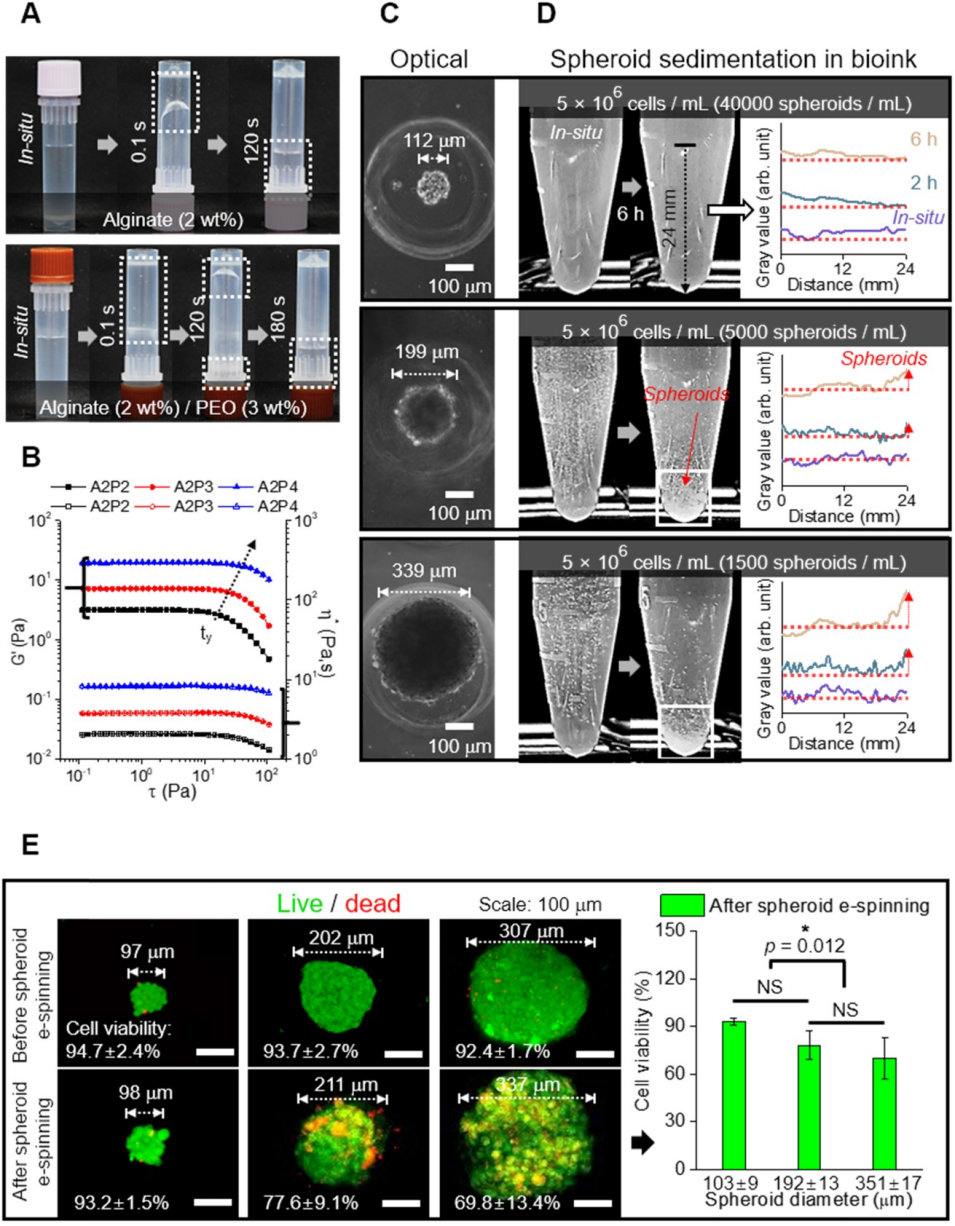
(A) Optical images showing relative viscose property of pure alginate (2 wt%) and alginate (2 wt%)/PEO (3 wt%) (A2P3) solution. (B) Rheological property (G' and η^*^) for stress sweep test and τ_y_ indicating yield stress. (C) Optical images of spheroid fabricated with different diameters (112, 199, and 339 μm); (D) Spheroid sedimentation for 6 h, gray values refer to the settled spheroids; (E) Live (green)/dead (red) images and viability of spheroids before and 6 hours following fabrication (n = 5).

In the alginate solution, we accommodated PEO as a supporting material because it can act not only as an electrospinning supplementary material, but also as viscosity-increasing material to avoid sedimentation of microscale spheroids. Figures 2A and B show the optical images indicating relative viscose property (at 23 °C) and rheological properties (G': storage modulus and η^*^: complex viscosity) for the stress (τ) sweep of the alginate (2 wt%)/PEO (2, 3, 4 wt%) solution, respectively. As shown in the optical images and rheological results, the addition of PEO in the alginate solution clearly increased the viscosity. Furthermore, the yield stress (τ_y_), an important criterion of the resistance force in hydrogel solution against the gravitational force of particles, in the alginate/PEO solutions was clearly increased as increase of PEO wt%. Thus, the addition of PEO can reduce the sedimentation of spheroids for electrospinning process. As considering the electrospinning ability 21 and yield stress of the alginate/PEO solution, we used the alginate (2 wt%)/PEO (3 wt%) (A2P3) bioink in this work.

To identify the appropriate size of spheroid for electrospinning, spheroids of sizes 112, 199, and 339 μm were used (Figure 2C). Spheroid diameter has been known to affect cell viability and metabolic activities, making it a significant factor in the construction of a scaffold 33, 34. For instance, utilization of hepatocytes revealed that cell viability decreased with increasing spheroid size (100~600 μm), while the specific rate of albumin secretion was the highest for spheroid with diameter 100 μm 33.

To investigate spheroid diameter with respect to compatibility with spheroid electrospinning, the number of spheroids with the same cell density (5 × 10^6^ cells/mL) to be put into the bioink (A2P3) was determined (Figure 2D). The bioinks, with homogeneously distributed spheroids, were then put into a microtube to observe the sedimentation overtime. The 199 and 339 μm spheroids began to settle after 2 h, and most of them were settled after 6 h. Meanwhile, the 112 μm spheroids revealed no significant change after 6 h. This implied that a syringe pump could uniformly supply 112 μm spheroids without precipitating the spheroids during the fabricating process (until 6 h).

Furthermore, the spheroids with different diameters (about 100 ~ 300 μm) were e-spun to observe *in situ* cell viability. In Figure 2E, the live (green) and dead (red) cells are shown, which are before and after e-spun with the processing condition (E = 0.075 kV/mm, 0.25 mL/h, and 3 min). Before electrospinning, the cell viability was high for all spheroids. After the electrospinning, the cell viability for 98 μm spheroids was high (>90%) and not significantly different from that for 211 μm spheroids. However, a significant decrease in cell viability was observed for the e-spun 337 μm spheroids due to increased electrostatic force (F), F = (μ•∇)E (where μ is dipole moment, which is proportional to the volume of particles, and E is applied electric field), acting on the particles in a non-uniform electric field (∇E ≠0) 35. In brief, when deposited on a collector, the bigger spheroids (≈larger volume) experienced greater electrostatic force, resulting in a much higher momentum compared to that of spheroids with smaller volumes. Based on the experimental results, we fixed the spheroid diameter, about 100 μm, for the spheroids-electrospinning process.

**Figure 3:**
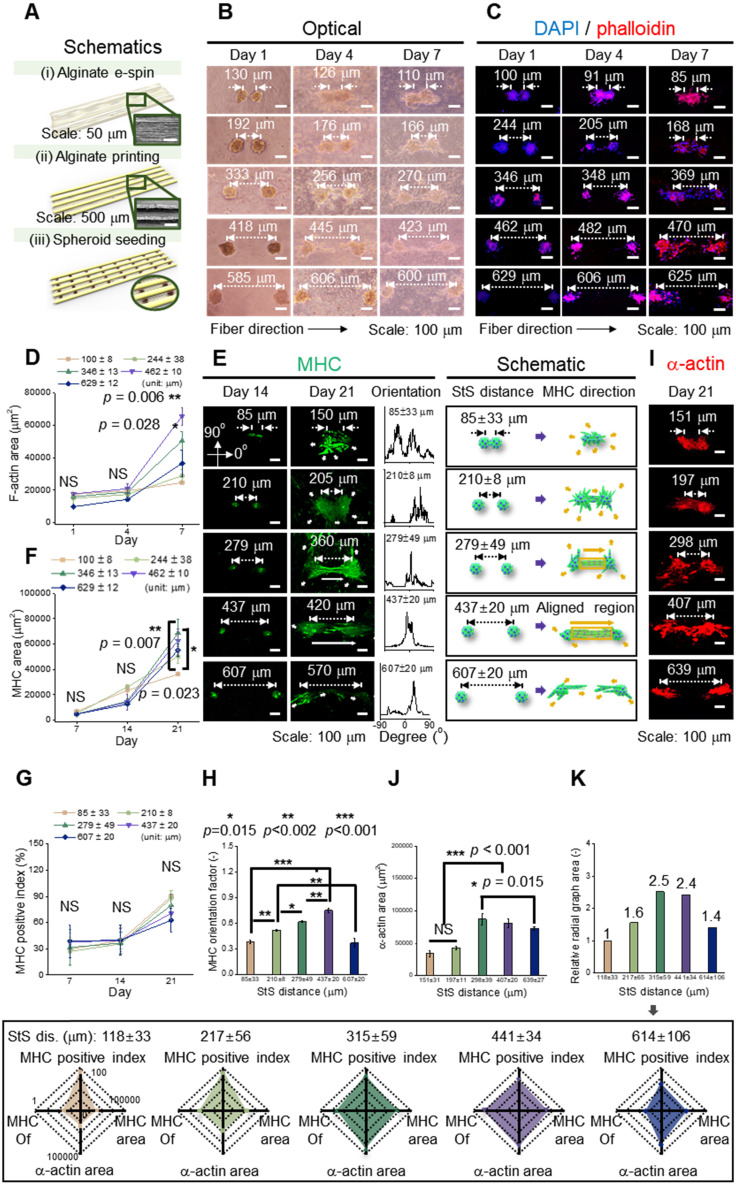
(A) Schematic representation of fabrication of a hybrid alginate structure that comprises aligned alginate fibers and parallel alginate struts; (B) Optical and (C) DAPI/phalloidin images of spheroids seeded on the hybrid structure at 1, 4, and 7 d; (D) F-actin area obtained from DAPI/phalloidin images (n = 6); (E) Myosin heavy chain (MHC) images of spheroids at different spheroid-to-spheroid distances and orientation distribution obtained from MHC images at 21 d and their schematic images; Analysis of MHC morphology with respect to (F) area (n = 6), (G) positive index (n = 30), and (H) orientation factor (n = 50); (I) Fluorescent images of sarcomeric α-actin and (J) analysis with respect to area (n = 6); (K) A summarized graph from the radial graphs indicating the degree of myoblast differentiation with respect to MHC positive index, MHC orientation factor, MHC area, and sarcomeric α-actin area.

We hypothesized that the e-spun spheroids (diameter = 103 ± 9 μm) could interact with each other in a fibrous environment, which would be affected by the distances between two spheroids. To test the hypothesis, an aligned alginate fibrous mat was fabricated [Figure 3A (i)]. Then, on the fibrous mat, parallel alginate struts were printed to form a groove-like structure, where spheroids could be seeded [Figure 3A (ii, iii)]. This was given to increase cell interactions in a uniaxial direction. The density of spheroids was adjusted to 1.9 × 10^5^, 1 × 10^5^, 6 × 10^4^, 4 × 10^4^, and 4 × 10^3^ spheroids/mL, and higher spheroid seeding density led to a shorter spheroid-to-spheroid (StS) distance. Next, the spheroids were observed at various target distances (118 ± 33, 217 ± 56, 315 ± 59, 441 ± 34, and 614 ± 106 μm).

Figure 3B and 3C shows the optical and fluorescent images (at 1, 4, and 7 d) in which spheroids were cultured on a fibrous mat in between the microgrooves. It represents the extension of spheroids, and fluorescent images (nuclei = blue and F-actin = red) present the branches of spheroids at 4 d and elongation and fusion at 7 d. To observe the development of F-actin at the distances between spheroids quantitatively, the F-actin area was measured, and as the distance was increased, the growth of F-actin accelerated. However, at the distance of 629 ± 12 μm, F-actin development decreased (Figure 3D). Based on the results, the different spheroid density determines the spheroid-to-spheroid distance, and consequently, 4 ± 10^4^ spheroids/mL was effective to induce spheroid elongation.

To detect the effects of the distances between the spheroids on myogenic activity, the MHC morphology was observed in experimentally and schematically (Figure 3E). As shown in the schematic images, the spheroids showed non-directional branches of MHC at the distances of 85 ± 33 or 210 ± 8 μm. Meanwhile, at 279 ± 49 or 437 ± 20 μm, lateral extension between the spheroids, indicated as an aligned MHC region, was observed. Further, the orientation distribution demonstrated non-directional MHC elongation at 85 ± 33 or 210 ± 8 μm and aligned MHC elongation at 279 ± 49 or 437 ± 20 μm. However, at relatively longer distances such as 607 ± 20 μm, the development of MHC was limited, even though each spheroid was extended in a direction parallel to that of the spheroids. From the results, we estimated that appropriate distance between the spheroids was required to attain efficient StS interaction.

The expression of MHC was characterized quantitatively to show the effects of StS distances. The MHC area was similar until 14 d, and the area was highest at a distance of 279 ± 49 μm at 21 d (Figure 3F). Meanwhile, the MHC positive index (the ratio of nuclei within an MHC area) was not significantly different among different StS distances, and the index above 60% was exhibited at all distances at 21 d (Figure 3G). However, the orientation factor, which = (90 - φ_o_)/90 where φ_o_ is the full width at half maximum, was distinct for each group, demonstrating better MHC alignment with increase in the StS distance, except at distance 607 μm (Figure 3H). We comprehended that the spheroids at 607 ± 20 μm could experience weak StS interactions.

The sarcomeric α-actin, an essential protein for muscle contraction, was observed at a mature stage of differentiation (Figure 3I). The greater sarcomeric α-actin area was observed at 298 ± 39 and 407 ± 20 μm StS distance, indicating that these spheroids, compared to those that were too close or too far away, were able to attain a higher degree of myogenic differentiation (Figure 3J). In detail, densely packed myoblasts tend to form unorganized cell clusters, which hamper cell alignment and differentiation 36. Meanwhile, a certain distance (>50 μm) is required for single myoblast cells to be able to interact and elongate each other 37, and the result suggests that two spheroids were able to elongate and differentiate most appropriately from 298 ± 39 to 407 ± 20 μm.

Based on the previous evaluation of the effect of StS distance on the development of MHC and sarcomeric α-actin, myoblastic activities was summarized and represented in a radial graph that included MHC positive index, MHC orientation factor, MHC area, and sarcomeric α-actin area (Figure 3K). As shown in the graphs, 315 ± 59 and 441 ± 34 μm StS distances, compared to the other StS distances, could provide an appropriate microcellular environmental condition for the spheroids. Hence, we selected the spheroid density, 4 × 10^4^ spheroids/mL, to obtain the range of the required StS distance, 441 ± 34 μm, for the spheroid-electrospinning process. Then, the same cell density (4 × 10^4^ spheroids/mL; 5 × 10^6^ cells/mL) used for spheroid-electrospinning was used for cell-electrospinning process.

### Electrospinning of spheroids

**Figure 4:**
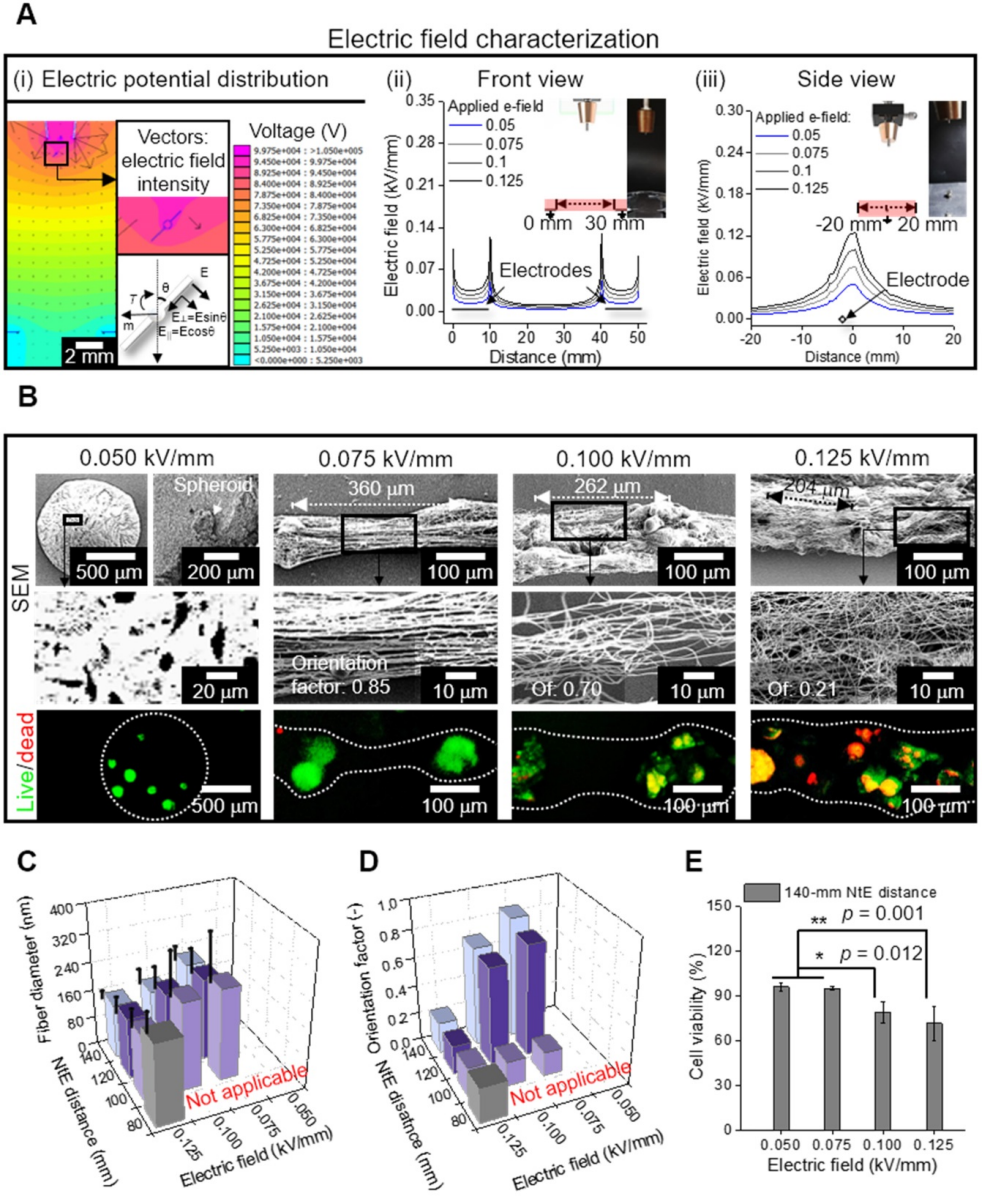
(A) (i) Electric potential distribution reproduced as a simulation, in which the spheroid-laden fiber is affected by field-induced torque; Electric field characterization of the area in the red box, which is illustrated in (ii) front and (iii) side views; (B) SEM and live/dead images of electrospun spheroid-laden structures in different electric fields (0.05~0.125 kV/mm); (C) Fiber diameter (n = 30) and (D) orientation factor (n = 50) for various processing conditions; (E) cell viability of e-spun spheroids (n = 5).

For the electrospinning solution, alginate was selected for non-toxic and rapid gelation of the cations (Ca^2+^), allowing versatile abilities of the process 38, 39. Furthermore, PEO was added to the alginate bioink as a process-supporting material, increasing the viscosity of the solution, and reducing surface tension and electrical conductivity 39, 40. These conditions were necessary for the reduction in repulsive force among the polyanionic alginate molecules and molecular chain entanglement, allowing stable fiber formation 41. For cell/spheroid electrospinning, the compositions of alginate (2 wt%) and PEO (3 wt%) [electrical conductivity = 3.23 ± 0.01 mS/cm, surface tension = 35 ± 5 mN/m] were selected from our previous study 21. Figure 4A (i) shows the electric potential distribution, which was simulated using the same dimension as that of the electrospinning set-up. The simplified spheroid-laden fiber was reproduced, in which the surrounding electric fields caused the torque to align fibers in a direction of the grounded parallel electrodes. To elaborate, the torque equation (≈μ×E) denotes that the rotating direction of the cell/spheroid-laden fibrous structure could be directly determined by calculating the field-induced torque 35, 42. Therefore, the electric field manipulated by parallel and grounded electrodes was utilized to generate aligned cell/spheroid-laden fibers.

The electric field intensity near the grounded electrodes was analyzed quantitatively with respect to the electrode-to-electrode (EtE) distance (Figure 4A (ii)). The simulated electric field showed two peaks at the end of the electrodes in the front view [Figure 4A (ii)]. These peaks became higher and broader as the applied electric field increased, indicating that higher the electric field applied to the process, more efficiently were the charged cell/spheroid-fibers aligned in the direction of the electric field. However, in the side view of the electrodes, as the applied electric field increased, a higher but broader distribution of the electric field was obtained, resulting in the charged fibers being deposited in broader regions [Figure 4A (iii)].

To evaluate the effect of the electric field experimentally, the spheroid-nanofiber formation was examined using various electric fields (0.05, 0.075, 0.1, and 0.125 kV/mm) (Figure 4B). The spheroid-laden bioink collected using an electric field of 0.05 kV/mm was droplet shaped because the electrostatic force was not sufficient for fiber formation. Furthermore, the spheroid-laden nanofibers were fabricated using an electric field of at least 0.075 kV/mm, and the Taylor cone was developed by whipping the fibers containing spheroids. In addition, the aligned spheroid-fibers were deposited between the electrodes, and as expected from electric field simulation (Figure 4A (i)), the deposited region of the spheroid-fibers gradually widened as the electric field increased from 0.075 to 0.125 kV/mm. Meanwhile, the StS distance decreased, which may hinder spheroid alignment 36. From the results, we estimated that the alignment of spheroid-fibers could be decreased on increasing the electric field.

Furthermore, the electric field directly affected cell viability of the cell/spheroid-laden fibers, and hence, we measured the cell viability of the spheroid. As shown in the live/dead images, we observed that the appropriate electric field with respect to cell viability could exist.

For more detailed characterization of the spheroid-fiber structure, various nozzle-to-electrode (NtE) distances (80~140 mm) and electric fields (0.05~0.125 kV/mm) were used in the processing diagram. The fiber diameter decreased with greater NtE distance and higher electric field (Figure 4C). Nevertheless, the alginate fibers fabricated in the electro-spinnable range were all in nanoscale. The orientation factor of the e-spun fibers was high at certain NtE distances (120~140 mm) and electric fields (0.075~0.1 kV/mm) (Figure 4D). Since 140 mm NtE distance revealed the highest orientation factor, we selected 140 mm distance to examine cell viability against various electric fields. The cell viability was high (>90%) when electric fields of 0.05 and 0.075 kV/mm were used and dropped significantly above 0.1 kV/mm (Figure 4E). For the highest orientation factor and reasonable cell viability, we selected 140 mm NtE distance and 0.075 kV/mm electric field.

### Fabrication of C2C12 single cell-laden and spheroid-laden structures

**Figure 5:**
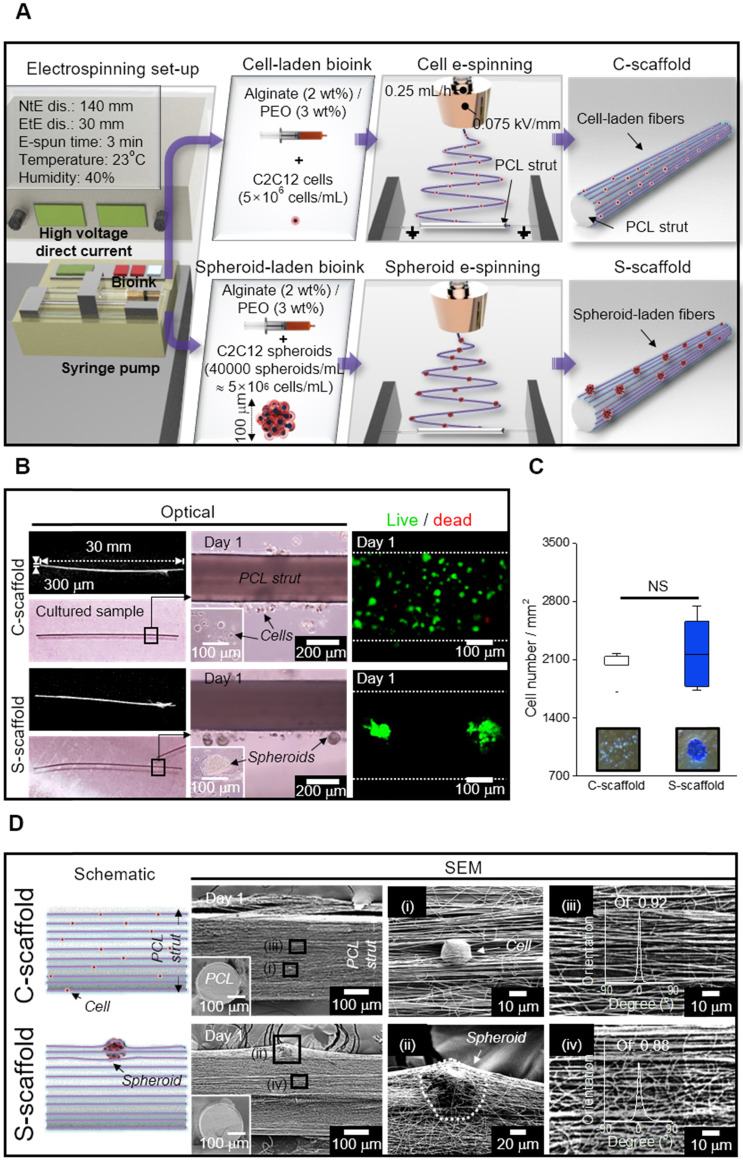
(A) Schematic representation of cell/spheroid electrospinning; (B) Cell-laden (C)-scaffold and Spheroid-laden (S)-scaffold, supplemented with a PCL strut, represented with optical and live/dead images; (C) Initial cell number measured from the optical images of Trypan blue assay (n = 3); (D) Schematic and SEM images of C-scaffold and S-scaffold in front view and cross-section (i, ii) the cell-/spheroid-laden fibers and (iii, iv) fiber orientation distribution.

As shown in the schematic image showing cell/spheroid electrospinning, the cell/spheroid-laden bioinks were e-spun onto a cylindrical PCL strut (diameter = 300 μm) mounted between parallel electrodes (Figure 5A). In this study, we denoted the arranged cell-laden fibrous structure and spheroid-laden structure as “C-scaffold” and “S-scaffold,” respectively.

Using the electrospinning conditions shown in Figure 5A, C-scaffold and S-scaffold were successfully fabricated as represented in optical and live/dead images (Figure 5B). In optical images of Figure 5B, a non-significant difference in diameter and height between S-scaffold and C-scaffold was observed (S-scaffold: 306.4 ± 7.6 mm (diameter) and 30.4 ± 0.7 mm (length); C-scaffold: 303.3 ± 7.3 mm (diameter) and 30.9 ± 0.8 mm (length)). Further, since the number of cells laden directly affected cellular activities, the number of e-spun cells was counted using Trypan blue assay, and the two groups (C-scaffold and S-scaffold) showed similar cell numbers (Figure 5C).

The surface and cross-sectional SEM images represented the cells/spheroids within the alginate nanofibrous structure generated by cell/spheroid-electrospinning [Figure 5D (i, ii)]. In addition, the fibrous structures magnified in Figure 5D (iii, iv) revealed a highly arranged fibrous array, where orientation factor was 0.92 and 0.88 for C-scaffold and S-scaffold, respectively. Herein, the cells/spheroids could sustain their cellular activities in the 3D fibrous structure, and the scaffolding structure could provide an aligned topological cue, as shown in the magnified SEM images.

### *In vitro* cellular activities on a hybrid structure

Figure S2A shows the optical and live/dead images of e-spun cells/spheroids cultured for 1 and 7 d. As shown in the optical images of cells cultured at 7 d, unlike the single cells of the C-scaffold, the spheroids in the fibers (S-scaffold) were spread in a direction similar to that of the aligned fibrous structure, as if they were pulling each other.

**Figure 6:**
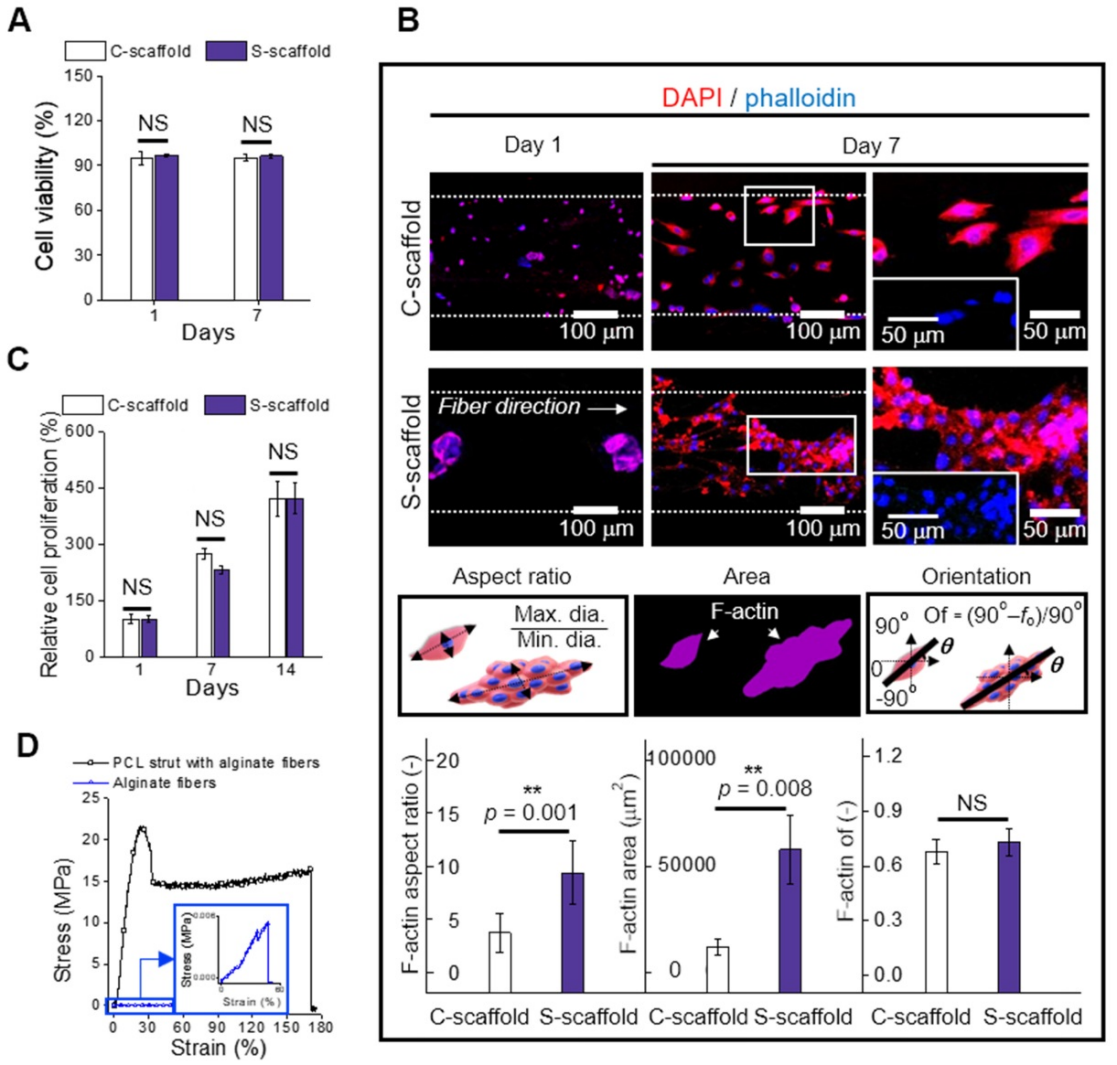
(A) Cell viability of cells and spheroids at 1 and 7 d (n = 5); (B) DAPI/phalloidin images and analysis of F-actin with respect to the aspect ratio (n = 30), area (n = 6), and orientation factor (n = 30) at 7 d; (C) Relative cell proliferation rate (n = 6) and (D) stress-strain curves of the PCL strut e-spun with alginate fibers and alginate fibers (n = 5).

To evaluate the effect of the applied electric field for the electrospinning process on cell viability, the number of live and dead cells of the cells/spheroids was measured. A high cell viability (>95%) of cells cultured for 1 and 7 d was observed, confirming that the fabricating process could be safely conducted for the e-spun cells/spheroids (Figure 6A). Furthermore, spheroid elongation and fusion were observed at 7 d, while the dispersed single cells began to form a fusiform shape during elongation.

The change in the cell/spheroid morphology was further evaluated using DAPI/phalloidin staining (Figure 6B). The circular shape of cells/spheroids was enlarged for 1 to 7 d, and typically, the spheroid deformed into a bumpy shaped structure, indicating breakage of the spherical shape by spreading of the spheroids. Motility of the spheroid indicated that the scaffolding fibrous structure was stable and reasonably cohesive for cellular activities 43. This spheroid-to-matrix interaction allowed spheroids to preferentially spread in an anisotropic manner into an aligned micro/nanopattern 44.

The change in the cell/spheroid morphology was analyzed to evaluate the aspect ratio, which is the ratio of maximum Feret's diameter to minimum Feret's diameter of the F-actin area (Figure 6B). The obtained aspect ratio for C-scaffold and S-scaffold at 7 d was “4 ± 2” and “9 ± 3”, respectively, revealing a 2-fold higher value for the S-scaffold. This denoted that elongation of the aggregated cells was achieved more efficiently on the S-scaffold than on the C-scaffold, and it could induce a more efficient formation of myotubes due to active cell-to-cell interaction. In addition, the F-actin area, which was 5-fold higher on the S-scaffold than on the C-scaffold, was evaluated. Meanwhile, the orientation factors of the C-scaffold (0.68 ± 0.06) and S-scaffold (0.73 ± 0.07) were similar, owing to the sufficient guidance of aligned e-spun fibers. In brief, these distinct cell morphologies could affect myogenesis of each cell/spheroid-laden structure in the fibrous structure.

Cell proliferation was measured at 1, 7, and 14 d, and normalized with the result of 1 d (Figure 6C, S2B). The C-scaffold and S-scaffold maintained an increasing rate of cell proliferation for 14 d, and no significant difference was observed, representing that the developed structure was biocompatible to support metabolic function of the cells/spheroids.

In this study, we accommodated the biocompatible PCL strut that can support the aligned cell/spheroid-laden fibrous structure. To evaluate mechanical stability, the stress-strain curves of the alginate fibers with and without supplementation of the PCL strut were obtained using a uniaxial tensile machine (Figure 6D). The tensile moduli were obtained using the initial slope and were 3631 ± 864 and 3 ± 1 kPa for alginate fibers with and without a PCL strut, respectively. The native muscle generally reveals 20 kPa of Young's modulus, which denotes the necessity of a mechanical supporter to enhance low mechanical property of alginate fibers 45. The stiffness of PCL was much higher than that of the alginate fibers, and PCL provided a stable microenvironment (topographical cue, etc.), which is essential as a mechanical supporter for cell growth and handling of the structure 21. Also, the PCL is biodegradable, so it has been applied for* in vivo* transplantation 46. Thus, the enhanced mechanical stability allowed C2C12 cells/spheroids to maintain various cellular activities for a longer cell culture period and practicability for transplantation.

### Differentiation and maturation of C2C12 cells and spheroids

**Figure 7:**
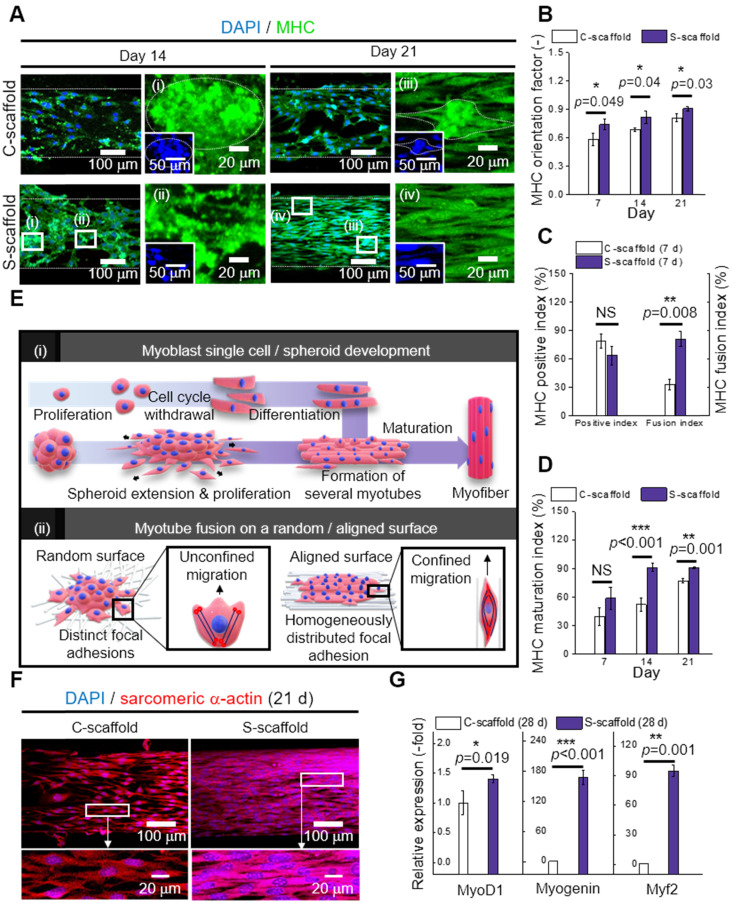
(A) DAPI/MHC images at 14 and 21 d; Analysis of MHC expression with respect to (B) orientation factor, (C) positive and fusion index, and (D) MHC maturation index (n = 30); (E) Schematic representation of (i) myoblast single cell/spheroid development and (ii) myotube fusion with a random/aligned topographical cue; (F) DAPI/sarcomeric α-actin images of C-scaffold and S-scaffold at 21; (G) Relative myogenic gene expression of MyoD1, myogenin, and Myh2 at 28 d (n = 5).

MHC is a motor protein of muscle tissue and was evaluated as an index for C2C12 differentiation. Hence, the images of MHC were captured at 14 and 21 d to observe the development of MHC (Figure 7A). Most cells/spheroids revealed MHC formation at 14 d, and as expected, the morphology of MHC further developed into multi-nucleated structures at 21 d. However, MHC development behavior of the C-scaffold and S-scaffold was different with respect to the formation rate of the condensed and elongated regions, which efficiently formed multi-nucleated myotubes for each structure. As shown in the MHC images at 14 d, the MHC region in the C-scaffold was dispersed and not concentrated, whereas the MHC region in the S-scaffold was condensed and elongated. The tendency to form MHC was clearly observed in a 21 d cell-culture. As shown in the DAPI/MHC images at 21 d, the MHC formation in the S-scaffold was significantly enhanced compared to that in the C-scaffold. Using the fluorescent images, the degree of MHC alignment was evaluated quantitatively as the orientation factor, which was greater for the S-scaffold throughout 21 d (Figure 7B). In addition, the MHC positive index was not different for the two groups at 7 d, but the MHC fusion index (the ratio of two or more nuclei within an MHC area) was higher for the S-scaffold (Figure 7C). Further, higher maturation index (the ratio of five or more nuclei within an MHC area) of the S-scaffold supported that MHC fusion and elongation was achieved more effectively by spheroid electrospinning (Figure 7D).

To explain the phenomenon briefly, the development of myoblast cells/spheroids is schematically shown in [Figure 7E (i)]. For myoblast single cell formation, after initiation of myogenic differentiation by myogenin or MRF4, the single myoblast lengthened in shape to form a myocyte. The myocytes fused with each other to form multinucleated myotubes, which expressed muscle specific proteins such as MHC or α-actin. Thereafter, several myotubes formed and matured to form a myofiber.

However, the myoblast spheroids underwent differentiation and fusion under external stimulation (aligned nanofibers) simultaneously with less time consumed due to the more efficient cell-to-cell interaction. Hence, myogenic differentiation/maturation occurred more rapidly for the spheroid structure than for the single cells.

To evaluate cell-to-matrix interactions, myotube fusion was easily achieved on an aligned surface [Figure 7E (ii)]. The focal adhesions were homogeneously distributed when a cell perceived the aligned surface, and the cellular structures, such as F-actin and microtubules, were arranged parallel to the surface 47. Here, the microtubule dynamic played a critical role in confined migration, and its reduced directionality induced anisotropic migration of the cells. Briefly, this environmental factor could accelerate myotube fusion by rearranging the spheroid shape into an elongated structure.

The sarcomeric α-actin was a typical structure found in multinucleated myotubes with Z line and α-actin. Hence, the development of sarcomeric α-actin indicated the maturation of myotubes. To elaborate Figure 7F and 7G, the surface of the S-scaffold is covered entirely with sarcomeric α-actin. On the other hand, the C-scaffold represented the elongated, but not multinucleated sarcomeric α-actin structure. It can be comprehended that a proper distance between spheroids allowed spheroid elongation and fusion 48. For longer culture periods, S-scaffold revealed fused myoblasts in line at 28 d and a striated pattern at 35 d, while C-scaffold did not reveal the pattern (Figure S1B, C). These results indicated that the e-spun spheroids greatly induced myotube formation and maturation.

Real-time PCR was used to examine myogenic gene expression at 28 d (Figure 7G). MyoD is a regulatory factor of myogenesis, and it was 1.4-fold higher for the S-scaffold. Myogenin, a factor that is expressed in the late mitotic stage, was 168-fold higher for the S-scaffold. Consequently, higher value of Myh2, comprising MHC and assisting in muscle contraction, was obtained for the S-scaffold (95-fold). In short, the myogenic gene expression was greatly increased for the S-scaffold, showing that mature myotubes were developed by synergistic effects of cell-to-cell/matrix interactions.

### Spheroid electrospinning using various cell types

To extend the effectiveness of the processing method for another cells, HUVEC spheroids were electrospun with the same composition of alginate bioink. The same electrospinning conditions, which were attained in the process of the e-spun C2C12-spheroids, were applied in the HUVEC-spheroid/alginate bioink, and the cell viability of the HUVECs was 92.5 ± 1.7% (sample size: 304.9 ± 6.4 mm (diameter) and 30.2 ± 0.9 mm (length)). After culturing the e-spun spheroids, the fluorescence images of CD31 and VE-cadherin were observed in the groups (cell-seeding group, e-spun single cells, and e-spun spheroids) (Figure S3). As seen in the images, the e-spun HUVEC-spheroids cultured for 14 and 21 days showed significantly higher cell proliferation and higher expression of CD31 (green) demonstrating fully developed vessel networks compared to the other groups. Furthermore, through the 3D image of CD31 and VE-cadherin, we can clearly observe the lumen formation in the group, e-spun spheroids. The integration of HUVEC spheroids is an evidence that nanofibers drove spheroids to elongate in a direction. Based on the results, we believe that the highly efficient cell-to-cell interactions caused by the e-spun spheroids can induce significantly higher cell-specific bioactive responses.

In addition, human muscle progenitor cells (hMPCs) were evaluated for cell/spheroid electrospinning. After the electrospinning process, high cell viability was observed on single cells (92.3 ± 3.5; sample size: 307.4 ± 5.8 mm (diameter) and 30.3 ± 0.5 mm (length)) and spheroids (95.6 ± 1.8 %; sample size: 306.3 ± 6.5 mm (diameter) and 30.9 ± 0.7 mm (length)). In the MHC images at 21 d, the hMPC spheroids revealed more elongated morphology of MHC with broader area compared to hMPC single cells, while the orientation factor was comparable (Figure S4). Also, the expression of sarcomeric a-actin was enhanced on hMPC spheroids. The cell growth and differentiation of hMPCs were supported by spheroid electrospinning, which has shown the possibility in clinical applications.

## Conclusion

This study suggested a new strategy to obtain a novel *in vitro* model that is utilized with uniaxially elongated spheroids comprising different cell types. To achieve the goal, various materials and processing parameters including the appropriate spheroid-distance for inducing C2C12-spheroid alignment and differentiation were investigated. By using the selected parameters, the spheroid-laden bioink was e-spun to obtain reasonable cell viability (>90%) and a highly arranged alginate nanofibrous structure. The developed fibrous structure was supplemented with a PCL strut, in which a stable culturing environment was provided for e-spun cells and spheroids to compare *in vitro* cellular activities. Consequently, the spheroid-laden structure greatly promoted myotube alignment, fusion, and maturation due to the synergistic effects of enhanced cell-to-cell/cell-to-matrix interactions, and its effectiveness was confirmed by enhanced myogenic gene (MyoD1, myogenin, and Myh2) expression. We believe that the spheroid-electrospinning can be an effective tool in inducing a high degree of myoblast differentiation and maturation, and the method using HUVEC-spheroids can be potentially performed as an efficient vascularization niche for angiogenesis to enhance tissue development *in vitro*. As a long-term application, the method can be expanded for various musculoskeletal tissues, such as tendons and ligaments, and applied for attaining an *in vitro* tissue-on-a-chip model in various drug testing.

## Supplementary Material

Supplementary figures.
