# Supplementary material for: An in vitro model using spheroids-laden nanofibrous structures for attaining high degree of myoblast alignment and differentiation
Source: Theranostics. 2021 Jan 16;11(7):3331–47. doi: 10.7150/thno.53928 (PMC7847672; doi:10.7150/thno.53928)
Supplement: Supplementary file 1 — Supplementary figures. [file thnov11p3331s1.pdf]

Supplementary Information

An *in vitro* model using spheroids-laden nanofibrous structures for attaining high degree of myoblast alignment and differentiation

Miji Yeo[1], SooJung Chae[1], GeunHyung Kim[1,2,*]

[1]Department of Biomechatronic Engineering, College of Biotechnology and Bioengineering, Sungkyunkwan University (SKKU), Suwon 16419, South Korea
[2]Biomedical Institute for Convergence at SKKU (BICS), Sungkyunkwan University, Suwon 16419, South Korea

[1]The authors contributed equally.

[*]Corresponding author
Professor GeunHyung Kim
Department of Biomechatronic Engineering, College of Biotechnology and Bioengineering, Sungkyunkwan University (SKKU), Suwon 16419, South Korea
E-mail: gkimbme@skku.edu; Tel.: +82-31-290-7828

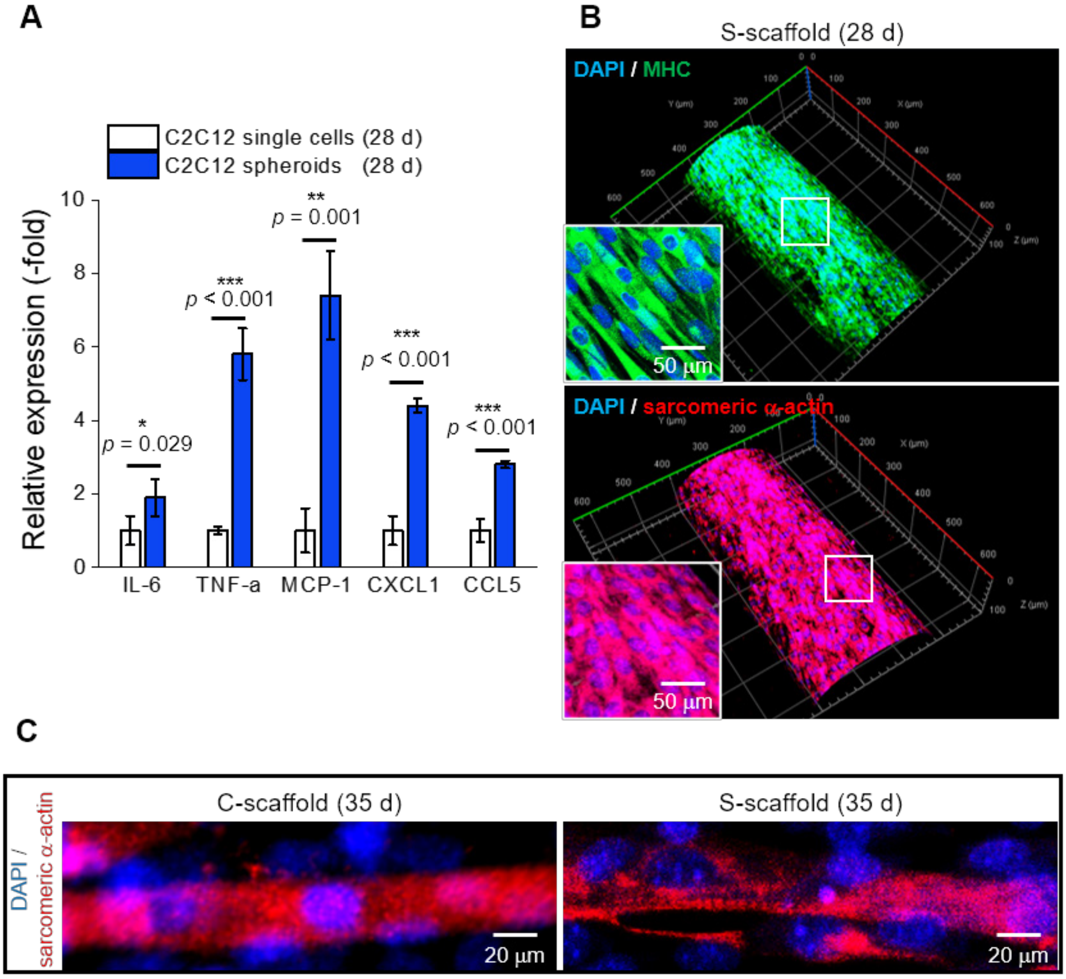

Figure S1. (A) Relative gene expression of the samples with electrospun C2C12 single cells/spheroids at 28 d (n = 5). (B) MYC and sarcomeric α-actin of S-scaffold at 28 d. (C) Sarcomeric α-actin of C-scaffold and S-scaffold at 35 d.

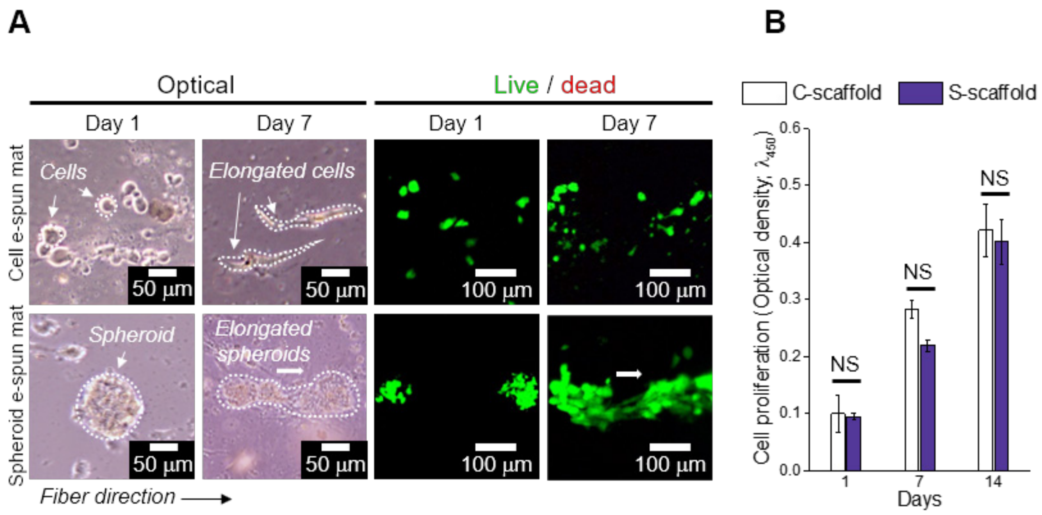

Figure S2. (A) Optical and live/dead images of cell e-spun and spheroid e-spun mat. (B) Optical density of cell proliferation for C-scaffold and S-scaffold.

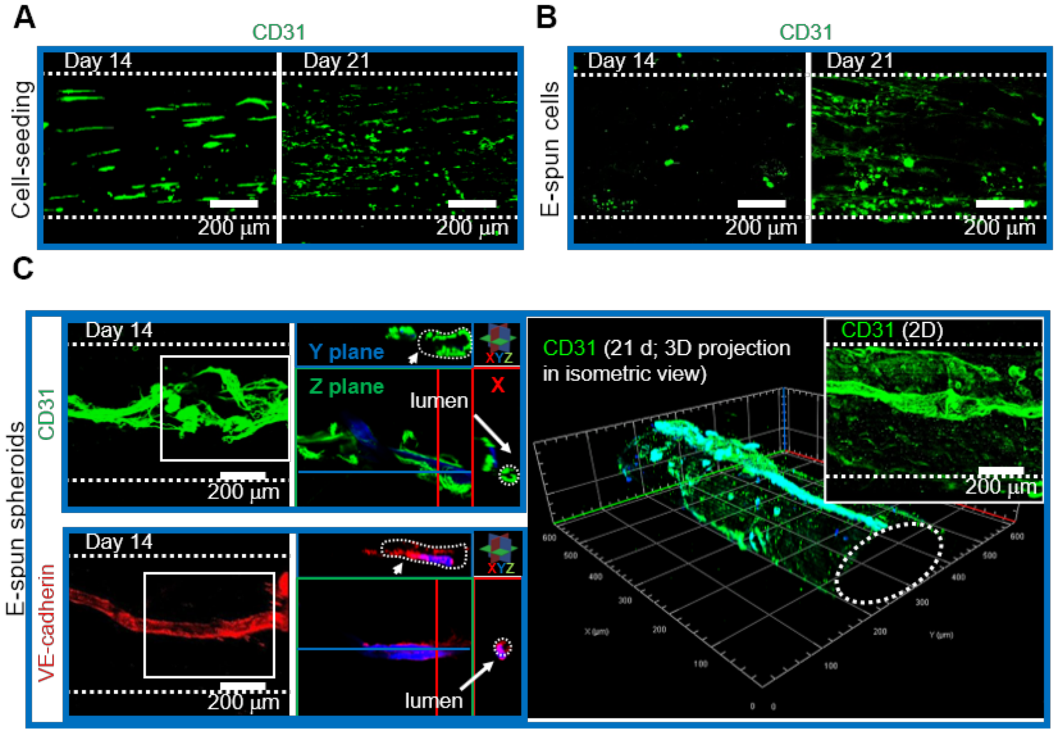

Figure S3. Fluorescence images of CD31 for (A) cell-seeded HUVECs and (B) cell-electrospun HUVECs at the culture-periods, 14 and 21 d. (C) Fluorescence images of CD31 (green) and VE-cadherin (red) of the electrospun HUVEC-spheroids at 14 and 21 d.

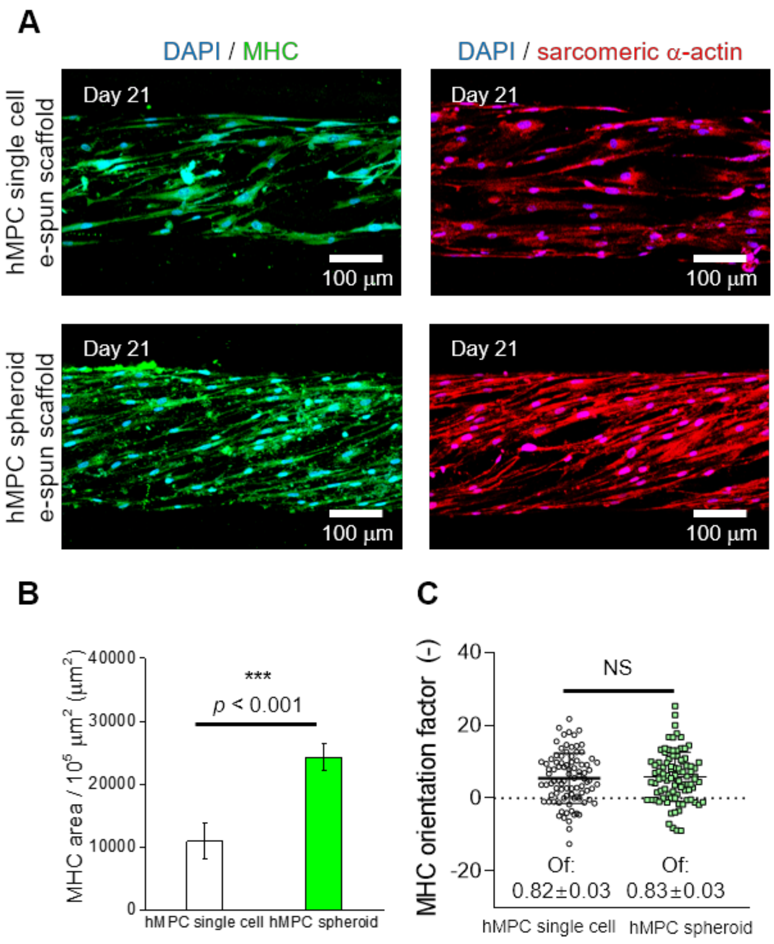

Figure S4. (A) Fluorescence images of MHC and sarcomeric α-actin for electrospun hMPC single cells and spheroids at 21 d. Analysis of MHCs with respect to (B) area and (C) orientation factor.
